# Diagnostic value of high‐risk HPV other than type 16/18 in high‐grade cervical neoplasia among cytology‐negative women: A multicenter retrospective study

**DOI:** 10.1002/cam4.6109

**Published:** 2023-05-18

**Authors:** Anying Bai, Peng Xue, Qing Li, Yu Jiang, Youlin Qiao

**Affiliations:** ^1^ Department of Epidemiology and Biostatistics, School of Population Medicine and Public Health Chinese Academy of Medical Sciences and Peking Union Medical College Beijing China; ^2^ Diagnosis and Treatment for Cervical Lesions Center Shenzhen Maternity & Child Healthcare Hospital Shenzhen China

**Keywords:** cervical cancer, cytology, HPV genotype, unnecessary referrals

## Abstract

**Background:**

Human papillomavirus (HPV) is a necessary cause of cervical cancer, and a tool more sensitive than cytology for the early screening of cervical precancers. The two most carcinogenic genotypes HPV 16/18 have been reported in the majority of studies. High‐risk HPVs other than HPV 16/18 (non‐16/18‐hrHPVs) cause approximately a quarter of cervical cancers, and we aimed to analyze the genotype‐specific prevalence, risk and diagnostic efficiency of non‐16/18‐hrHPVs in cervical carcinogenesis among Chinese cytology‐negative women.

**Methods:**

A total of 7043 females who had abnormal cervical testing results from January 2018 to October 2021 were enrolled, among them 3091 were cytology‐negative. Descriptive statistics was used to estimate the HPV genotype‐specific prevalence, and multivariable logistic regression was used to estimate the genotype‐specific non‐16/18 hrHPVs risk of cervical carcinogenesis. The evaluation of diagnostic value among HPV genotypes included the possibility of predicting cervical intraepithelial neoplasia grade 2/3 or worse (CIN2+/CIN3+) and the diagnostic efficiency measured by increased referral rate and referral numbers of colposcopies per additional CIN2+/CIN3+ detected.

**Results:**

Among HPV‐positive cytology‐negative women, the five dominant genotypes for CIN2+/CIN3+ were HPV 31/33/35/52/58. HPV 52/58/33 had comparatively high sensitivity and specificity in predicting CIN2+/CIN3+, while the referral strategy of multiple HPV58 required 26 colposcopies to detect 1 CIN3+, compared with 14, 12, and 8 required by multiple HPV52, 31, and 33, respectively.

**Conclusions:**

HPV31/33/35/52/58 infections are significant risk factors for cervical lesions, and multiple HPV 31/33/52 infections should be included in the previously recommended HPV16/18 genotyping triage for colposcopy in China, as the benefits of disease prevention may outweigh the disadvantages of increasing requirements for colposcopy services.

## 1 INTRODUCTION

Human papillomavirus (HPV) is a common sexually transmitted infection and is the cause of cervical cancer.^1^ In 2020, there were an estimated 604,000 new cases and 342,000 deaths from cervical cancer worldwide,^2^ with China having a high incidence of 7.5/100,000 and a mortality of 3.4/100,000 for cervical cancer, and an HPV infection rate of 16.8%.^3^ The US Preventive Services Task Force recommends directly referring women infected with HPV16/18 for coloscopy. Non‐16/18 hrHPV women would be triaged for cytology,^4^, ^5^, ^6^ while only cases of positive cytology could be identified through this approach. Given the relatively varied sensitivity of cytologic screening,^7^, ^8^ current guidelines increase the number of related missed opportunities to intervene earlier in the course of this insidious disease.

Managing women with discordant co‐testing, defined as HPV‐positive cytology‐negative (HPCN) remains a challenge. The prevalence of HPCN ranged from 1.9% to 9.8% in women over 30 years old, more than twice that with positive HPV and cytology among the same age population.^9^ Under current guidelines,^10^, ^11^ the follow‐up risk of HPCN women infected with non‐16/18‐hrHPVs might be prolonged,^12^, ^13^ resulting in increased pressure on finite medical resources and impacts on women's sense of well‐being. Moreover, cervical high‐grade lesions (about 10%) caused by non‐16/18‐hrHPVs might be missed,^14^, ^15^ particularly in areas with limited proficient cytologists like China.

Exploring the most carcinogenic types is beneficial, and may provide a basis for developing more in‐depth preventive strategies for HPCN women as the genotype‐specific prevalence and risk of the non‐16/18‐hrHPV remained inconclusive in China currently.^16^ Therefore, this study aimed to explore the prevalence, risk, and diagnostic efficacy of non‐16/18‐hrHPV genotypes among HPCN women from a large Chinese population in six provinces.

## 2 METHODS

### 2.1 Study population

**FIGURE 1:**
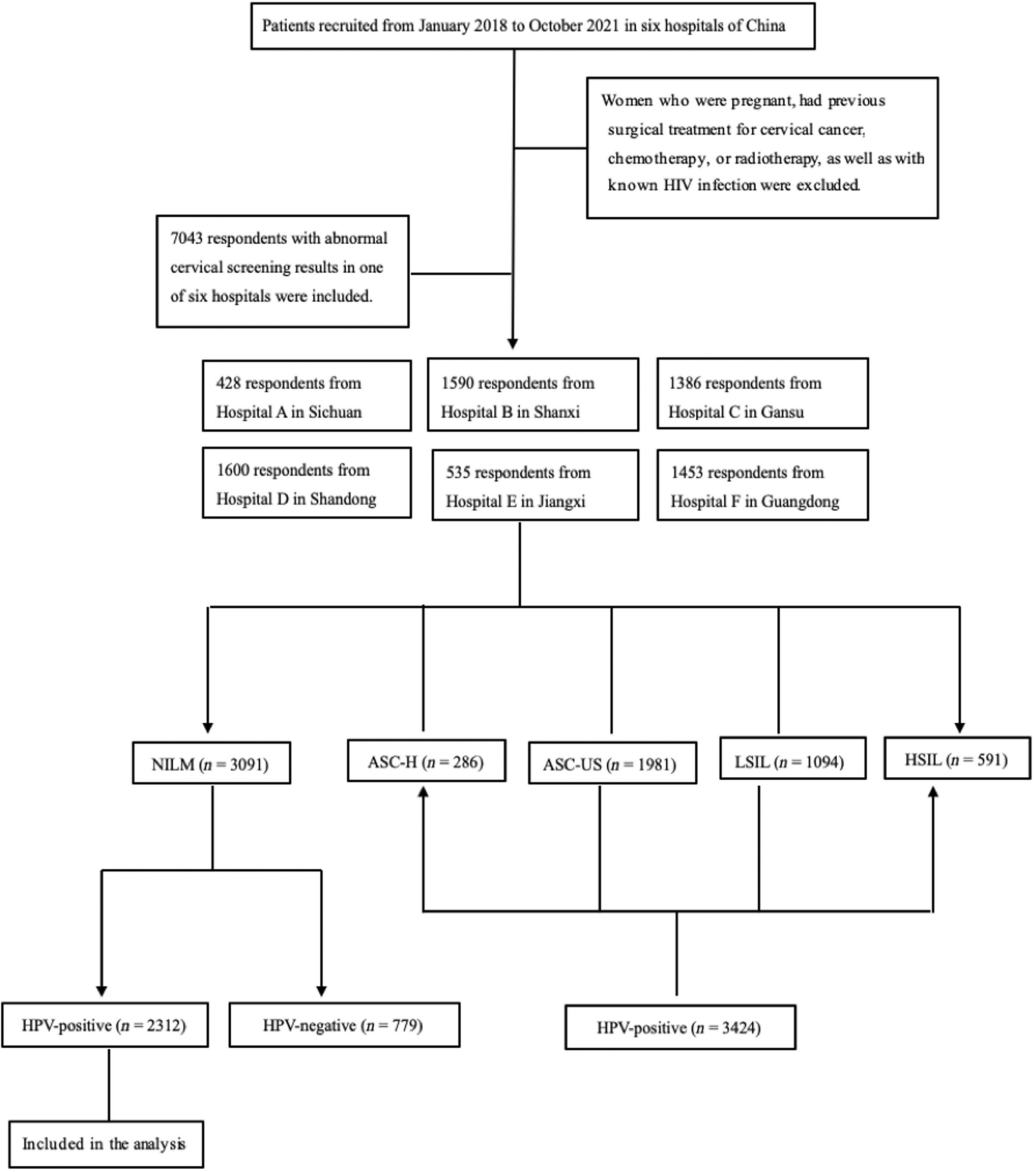
Flowchart of participant selection.

Patients from one of six hospitals in Sichuan, Shanxi, Gansu, Shandong, Jiangxi, and Guangdong provinces of mainland China in the department of Diagnosis and Treatment Centre of Cervical Diseases from January 2018 to October 2021 were initially selected to participate. Among them, women who were pregnant, had previous surgical treatment for cervical cancer, chemotherapy or radiotherapy, as well as with known HIV infection were excluded. Furthermore, remaining patients with abnormal cervical screening results and complete demographics and clinical information were selected. Among the 7034 selected patients, 3091 women with negative cytology results and complete HPV status information were included to evaluate the genotype‐specific prevalence, risk and diagnostic efficiency of non‐16/18‐hrHPVs (Figure 1).

This study was conducted in accordance with the Declaration of Helsinki and received ethical approval from the Institutional Review Board (IRB) at all hospitals. The need for informed consent was waived since the study was retrospective and personal information was anonymized.

### 2.2 Cytology and HPV detection

Thinprep cytologic test (TCT, Hologic) was used to perform cytologic analysis. Results were categorized into five classes according to the Bethesda System: negative for intraepithelial lesion or malignancy (NILM), atypical squamous cells of undetermined significance (ASCUS), atypical squamous cells–cannot exclude high‐grade squamous intraepithelial lesion (ASC‐H), low‐grade squamous intraepithelial lesion (LSIL), and HSIL and squamous cell carcinoma (HSIL/SCC). Computer‐assisted imaging was not used in the cytologic analysis.

HPV genotyping detected from liquid‐based cytology specimens collected were conducted afterward. HPV GenoArray test kit (HybriBio Ltd) approved by CFDA were used. This kits is capable of identifying 14 high‐risk HPV (HR‐HPV) types: 16, 18, 31, 33, 35, 39, 45, 51, 52, 53, 56, 58, 59, and 68. Patients infected with hrHPV types other than HPV16/18 were classified into “non‐16/18‐hrHPV” group. We also classified infected patients into three groups according to their HPV infected status: no infection, single infection and multiple infection. The risk among each HPV type in single or multiple infection of CIN2+ and CIN3+ was evaluated individually.

### 2.3 Coloscopy and biopsy protocol

General assessment was conducted in accordance with the 2011 IFCPC colposcopic terminology^17^ for the cervix which includes transformation zone types 1, 2, or 3. All colposcopies were performed by gynecologists using an electronic colposcope (Goldway Co. Limited).

Biopsies were taken using abnormal image sites and endorcervical curettage (ECC) was performed only when lesions under colposcopy were not visible in the cervical canal or squamacolumn junctions. 10% formaldehyde solution was used to fix, embed, and for tableting. Hematoxylin and eosin (HE) staining was then conducted before samples were sent to inspection. Biopsy specimens were sorted according to colposcopically defined severity, and two pathologists from the same hospitals read double‐blind films to record diagnosis for each quadrant. Where there were inconsistent comparisons, the histological pathology was determined by the senior pathologists or pathologists with more experience. Histopathological criteria were established according to the 4th edition of the 2014 WHO classification standard of female genital tumors, which includes negative results (no significant pathological findings, reactive or inflammatory processes, atypical squamous cell or glandular changes, or squamous metaplasia), CIN1, CIN2, CIN3, squamous cell carcinoma (SCC), and adenosquamous carcinoma (ASC). The latter four were compiled into one category referred to as “CIN2+” and the latter three were compiled into another category referred to as “CIN3+”.

### 2.4 Statistical analysis

Descriptive statistics of demographic characteristics were conducted. Distribution of hrHPVs and prevalence of CIN2+/CIN3+ were calculated to evaluate the correlation between different HPV infection status and cervical intraepithelial neoplasia among NILM patients. Two disease end points were assessed: CIN grade 2 or higher (CIN2+) and CIN grade 3 or higher (CIN3+).

Associations between combined non‐16/18‐hrHPVs among single or multiple HPV infection, and incidence of CIN2+/CIN3+ were examined using logistic regression models, adjusted for potential confounding by age, gravidity, parity, and menopause. Unconditional multivariable logistic regression was used to analyze the potential association between HPV genotype‐specific infection and cervical lesions. Odds ratio (OR) and 95% confidence intervals (95% CIs) were calculated.

In this study, to appraise resource utilization of triage scenario among HPCN women with non‐16/18‐hrHPVs, we assessed the diagnostic value of different non‐16/18‐hrHPVs genotyping models in detecting underlying CIN2+/CIN3+. First, diagnostic parameters (sensitivity, specificity, positive predictive value, negative predictive value, positive likelihood ratio, and negative likelihood ratio) of non‐16/18 hrHPVs infection were calculated to measure the clinical performance of each genotyping. Besides, compared with only triaging HPV 16/18 patients for coloscopy, the increased yields of CIN2+/CIN3+, referral number for colposcopy, as well as colposcopies per additional CIN2+/CIN3+ detected among non‐16/18 hrHPVs patients were also calculated as indicators of the diagnostic efficiency. Data management and statistical analysis were performed with STATA software version 16. p values were two‐sided, and statistical significance was accepted if the p value was 0.05 or less.

## 3 RESULTS

### 3.1 Characteristics of included patients

Table S1 provides demographics including age, gravidity, parity, menopause status and histopathology outcomes among 3091 women with NLIM, 286 with ASC‐H, 1981 with ASC‐US, 1094 patients with LSIL, and 591 patients with HSIL. The mean age of NILM group was 38.7 years, slightly lower than those in the other cytology groups. Patients from Hospital in Shandong constituted the highest percentage in all cytology groups, and the incidence of menopause in NLIM women were 13.9% higher than the 12.5% observed in LSIL cases. <CIN2 was the most prevalent among NILM women.

### 3.2 Prevalence of hrHPVs among cytologic‐negative women

**FIGURE 2:**
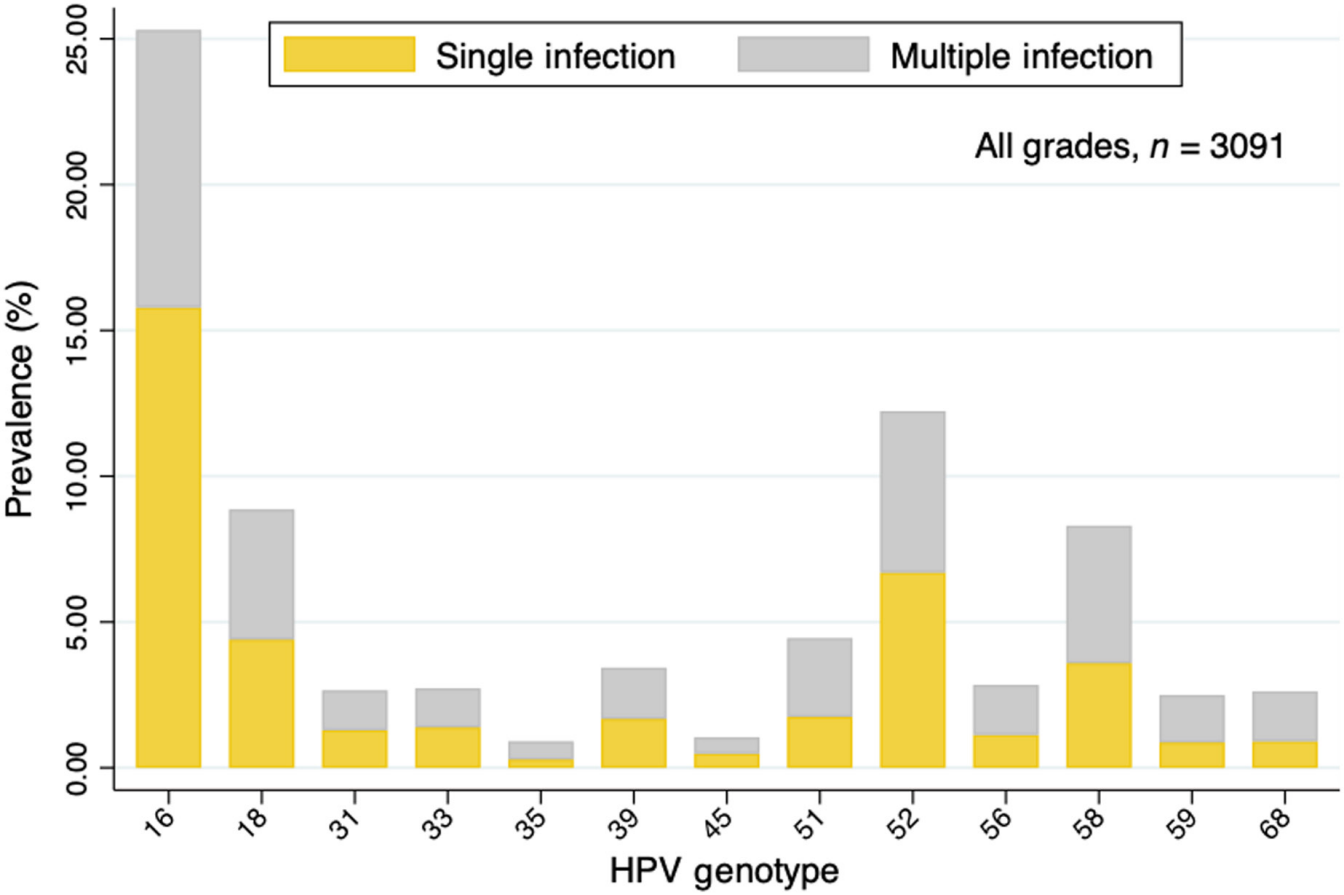
Prevalence of hrHPVs among cytologic‐negative women.

HPV single infections accounted for 52.86% of all cases, higher than multiple infections (28.58%). Among HPCN women, the frequencies of HPV subtypes besides HPV 16/18 were single HPV 52 infection (6.70%), multiple HPV 52 infection (5.53%), multiple HPV 58 infection (4.71%), single HPV 58 infection (3.59%), and multiple HPV 51 infection (1.75%). Other prevalence of HPV genotypes in HPCN women was shown in Figure 2.

### 3.3 Prevalence of CIN 2+/CIN 3+ among HPCN women

Of 3091 cytologic‐negative women, 2312 (74.87%) were HPV‐positive, 2672 (86.44%) had no visible CIN or CIN1, 298 (9.64%) had CIN2+, and 121 (3.91%) had CIN 3+. As shown in Table 1, the prevalence of double infection took up the most of multiple infection cases. Prevalence of CIN2+ was significantly higher among patients infected with HPV 31/33/52/68, and prevalence of CIN3+ was significantly higher among patients infected with HPV 33/35/45/52 compared with other infection status.

**TABLE 1:** Genotype‐specific prevalence of CIN 2+/CIN 3+ based on HPV‐infected status among HPCN women.

	Single infection (*n* = 1602)		Double infection (*n* = 409)		Triple infection (*n* = 248)		Multiple infection (*n* = 53)	
	No. CIN2+ (%)	No. CIN3+ (%)	No. CIN2+ (%)	No. CIN3+ (%)	No. CIN2+ (%)	No. CIN3+ (%)	No. CIN2+ (%)	No. CIN3+ (%)
HPV‐16	158 (32.38)	47 (9.63)	48 (25.40)	22 (11.64)	6 (10.17)	12 (20.34)	1 (2.38)	7 (16.67)
HPV‐18	16 (11.76)	5 (3.68)	12 (13.48)	6 (6.74)	3 (8.82)	0 (0.00)	3 (16.67)	0 (0.00)
HPV‐31	10 (25.00)	1 (2.50)	7 (50.00)	2 (14.29)	2 (12.50)	1 (6.25)	1 (8.33)	0 (0.00)
HPV‐33	10 (23.26)	3 (6.98)	9 (56.25)	5 (31.25)	1 (4.55)	0 (0.00)	0 (0.00)	0 (0.00)
HPV‐35	3 (33.33)	1 (11.11)	1 (16.67)	1 (16.67)	1 (14.29)	0 (0.00)	1 (14.29)	0 (0.00)
HPV‐45	0 (0.00)	0 (0.00)	2 (28.57)	1 (14.29)	1 (16.67)	0 (0.00)	0 (0.00)	0 (0.00)
HPV‐51	1 (1.85)	0 (0.00)	4 (12.50)	0 (0.00)	4 (10.81)	1 (2.70)	1 (7.69)	0 (0.00)
HPV‐52	29 (14.01)	4 (1.93)	20 (26.32)	7 (9.21)	17 (21.79)	8 (10.26)	3 (15.00)	0 (0.00)
HPV‐56	3 (12.00)	2 (5.71)	3 (8.57)	1 (4.00)	2 (11.76)	1 (5.88)	3 (27.27)	1 (9.09)
HPV‐58	17 (15.32)	2 (1.80)	12 (21.82)	3 (5.45)	6 (10.53)	1 (1.75)	3 (16.67)	0 (0.00)
HPV‐59	0 (0.00)	0 (0.00)	1 (5.26)	1 (5.26)	2 (11.11)	0 (0.00)	1 (7.69)	0 (0.00)
HPV‐68	1 (3.57)	0 (0.00)	4 (22.22)	2 (11.11)	5 (19.23)	2 (7.69)	1 (11.11)	0 (0.00)

### 3.4 HPV genotype‐specific risks of CIN 2+/CIN 3+ among HPCN women

Among HPCN women, women infected with single non‐16/18 hrHPVs were significantly associated with 3.32 times increased odds of CIN2+ and 4.11 higher odds of CIN3+ after adjustments (Table 2). Similarly, women infected with multiple non‐16/18 hrHPVs were significantly associated with 2.95 times increased odds of CIN2+ and 4.39 higher odds of CIN3+ after adjustments.

**TABLE 2:** HPV genotype‐specific risks of CIN 2+/CIN 3+ among HPCN women.

HPV genotype	NILM				NILM			
	CIN 2+		CIN 3+		CIN 2+		CIN 3+	
	OR (95% CI)	*p* value	OR (95% CI)	*p* value	OR (95% CI)	*p* value	OR (95% CI)	*p* value
	Single infection				Multiple infection			
Combined infection								
Non‐16/18 hrHPV subtypes	3.32^a^ (2.86–3.84)	0.000	4.11^a^ (3.32–5.09)	0.000	2.95^a^ (2.40–3.62)	0.000	4.39^a^ (3.11–6.20)	0.000
Individual infection								
HPV‐16	4.60 (3.59–5.89)	0.000	3.32 (2.22–4.97)	0.000	2.29 (1.62–3.22)	0.000	3.95 (2.34–6.68)	0.000
HPV‐18	1.26 (0.72–2.17)	0.411	1.17 (0.46–2.98)	0.738	0.64 (0.37–1.09)	0.100	0.57 (0.23–1.40)	0.220
HPV‐31	3.20 (1.54–6.66)	0.002	0.83 (0.11–5.93)	0.827	1.62 (0.76–3.45)	0.215	1.19 (0.34–4.14)	0.783
HPV‐33	2.91 (1.41–6.01)	0.004	2.34 (0.70–7.80)	0.166	1.67 (0.79–3.54)	0.18	2.76 (1.00–7.60)	0.050
HPV‐35	4.80 (1.19–19.38)	0.027	3.90 (0.48–31.73)	0.203	0.92 (0.24–3.40)	0.891	1.11 (0.13–9.47)	0.922
HPV‐39	0.59 (0.18–1.91)	0.376	0.61 (0.08–4.51)	0.630	0.88 (0.40–1.94)	0.749	0.29 (0.04–2.24)	0.237
HPV‐45	4.60 (3.59–5.89)	0.000	3.32 (2.22–4.97)	0.000	0.66 (0.18–2.45)	0.540	0.51 (0.06–4.10)	0.523
HPV‐51	0.18 (0.03–1.32)	0.092	0.18 (0.03–1.32)	0.092	0.69 (0.34–1.39)	0.299	0.36 (0.08–1.58)	0.178
HPV‐52	1.56 (1.02–2.39)	0.092	1.56 (1.02–2.39)	0.092	1.79 (1.18–2.70)	0.006	1.99 (1.05–3.78)	0.035
HPV‐53	0.17 (0.02–1.25)	0.082	0.17 (0.02–1.25)	0.082	0.50 (0.26–0.99)	0.048	0.32 (0.08–1.36)	0.123
HPV‐56	0.90 (0.27–2.97)	0.863	0.90 (0.27–2.97)	0.863	0.90 (0.41–1.98)	0.796	0.97 (0.29–3.30)	0.967
HPV‐58	1.74 (1.01–2.98)	0.045	1.74 (1.01–2.98)	0.045	1.01 (0.61–1.69)	0.956	0.65 (0.25–1.70)	0.383
HPV‐68	0.36 (0.05–2.63)	0.312	0.36 (0.05–2.63)	0.312	1.11 (0.54–2.30)	0.773	1.29 (0.44–3.83)	0.645

^a^ Adjusted for age, gravidity, parity, and menopause.

Among HPCN women, compared with the uninfected group, higher risk for CIN 2+ was found with single HPV16 (OR = 4.60; CI = 3.59–5.89; *p* < 0.001), HPV33 (OR = 3.20; CI = 1.54–6.66; *p* = 0.002), HPV33 (OR = 2.91; CI = 1.41–6.01; *p* = 0.004), HPV 35 (OR = 4.80; CI = 1.19–19.38; *p* = 0.027), and HPV58 (OR = 1.74; CI = 1.01–2.98; *p* = 0.045), as well as multiple HPV16 (OR = 2.29; CI = 1.62–3.22; *p* < 0.001) and HPV52 (OR = 1.79; CI = 1.18–2.70; *p* = 0.006) infection.

Compared with the uninfected group, increased risk for CIN 3+ was found with single HPV16 (OR = 3.32; CI = 2.22–4.97; *p* < 0.001), multiple HPV16 (OR = 3.95; CI = 2.34–6.68; *p* < 0.001), HPV33 (OR = 2.76; CI = 1.00–7.60; *p* = 0.050), and HPV52 (OR = 1.99; CI = 1.05–3.78; *p* = 0.035).

Among the selected non‐16/18 hrHPVs genotyping based on genotype‐specific risk, the specificity of diagnosing CIN2+/CIN3+ showed good performance. HPV35/31/58 had relatively higher specificity compared with other genotypes. The detailed results are presented in Table 3.

**TABLE 3:** Performance of non‐16/18 hrHPVs infection in detecting CIN2+/CIN3+ among HPCN women.

Baseline positive	CIN2+						
	Sensitivity (%)	Specificity (%)	PPV (%)	NPV (%)	Accuracy (%)	LR+	LR−
HPV 52							
Single infection	6.23	93.50	23.30	75.80	49.85	0.96	1.00
Multiple infection	7.41	94.25	29.10	76.19	50.83	1.29	0.98
HPV 58							
Single infection	6.24	95.73	31.74	76.24	50.98	1.46	0.98
Multiple infection	6.88	95.55	32.96	76.33	51.21	1.55	0.97
HPV 31							
Single infection	2.24	98.67	34.86	76.03	50.45	1.68	0.99
Multiple infection	2.76	98.47	36.43	76.09	50.61	1.80	0.99
HPV 33							
Single infection	3.35	98.45	40.71	76.20	50.90	2.16	0.98
Multiple infection	3.35	98.52	41.91	76.21	50.94	2.27	0.98
HPV 35							
Single infection	0.53	99.00	28.12	75.88	50.05	1.23	1.00
Multiple infection	1.35	99.20	34.85	75.96	50.27	1.68	0.99
	CIN3+						
	**Sensitivity (%)**	**Specificity (%)**	**PPV (%)**	**NPV (%)**	**Accuracy (%)**	**LR+**	**LR−**
HPV 52							
Single infection	5.31	93.42	7.69	90.53	49.37	0.81	1.01
Multiple infection	6.07	93.84	9.24	90.64	49.96	0.99	1.00
HPV 58							
Single infection	5.46	95.33	10.78	90.71	50.40	1.17	0.99
Multiple infection	5.16	95.00	9.58	90.65	50.07	1.03	1.00
HPV 31							
Single infection	1.52	98.45	9.17	90.64	49.98	0.98	1.00
Multiple infection	1.97	98.18	10.08	90.66	50.08	1.09	1.00
HPV 33							
Single infection	4.40	98.26	20.71	90.87	51.33	2.53	0.97
Multiple infection	3.49	98.23	16.91	90.79	50.86	1.97	0.98
HPV 35							
Single infection	0.15	99.51	3.12	90.61	49.83	0.31	1.00
Multiple infection	0.30	99.00	3.03	90.58	49.65	0.30	1.01

Abbreviations: LR+, positive likelihood ratio, LR–, negative likelihood ratio; NPV, negative predictive value; PPV, positive predictive value.

**FIGURE 3:**
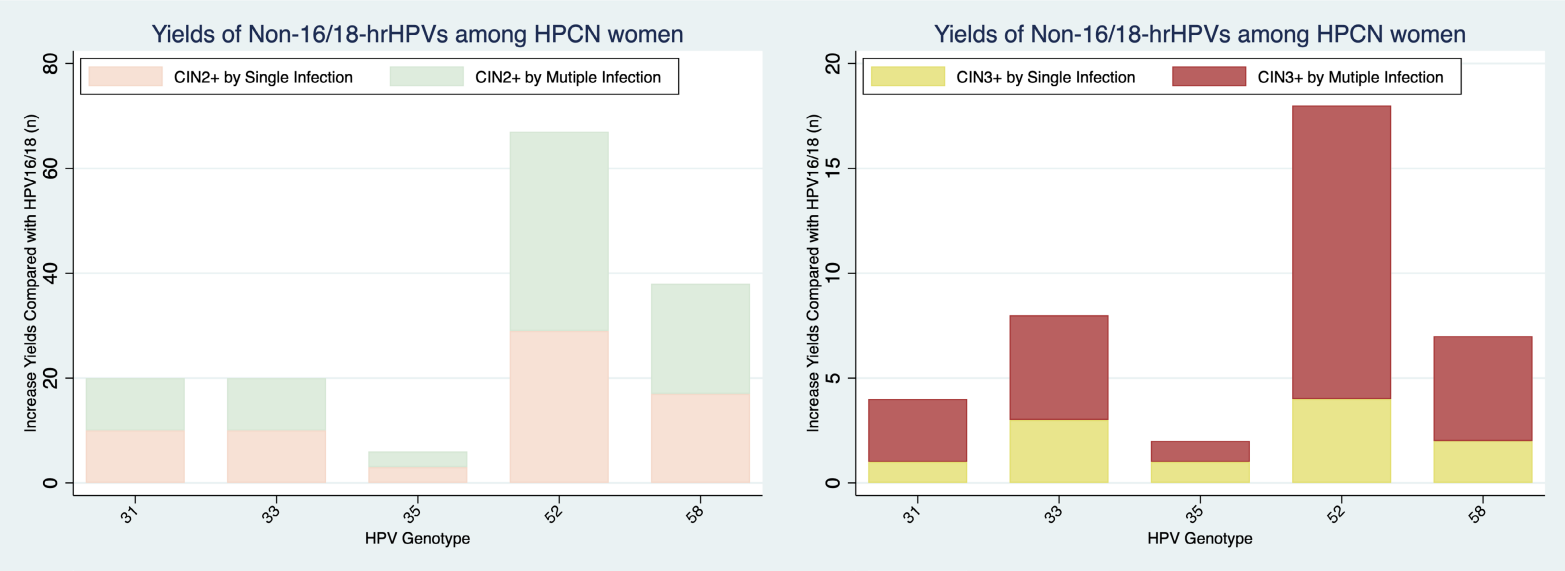
Yields of Non‐16/18 hrHPVs among HPCN women.

**FIGURE 4:**
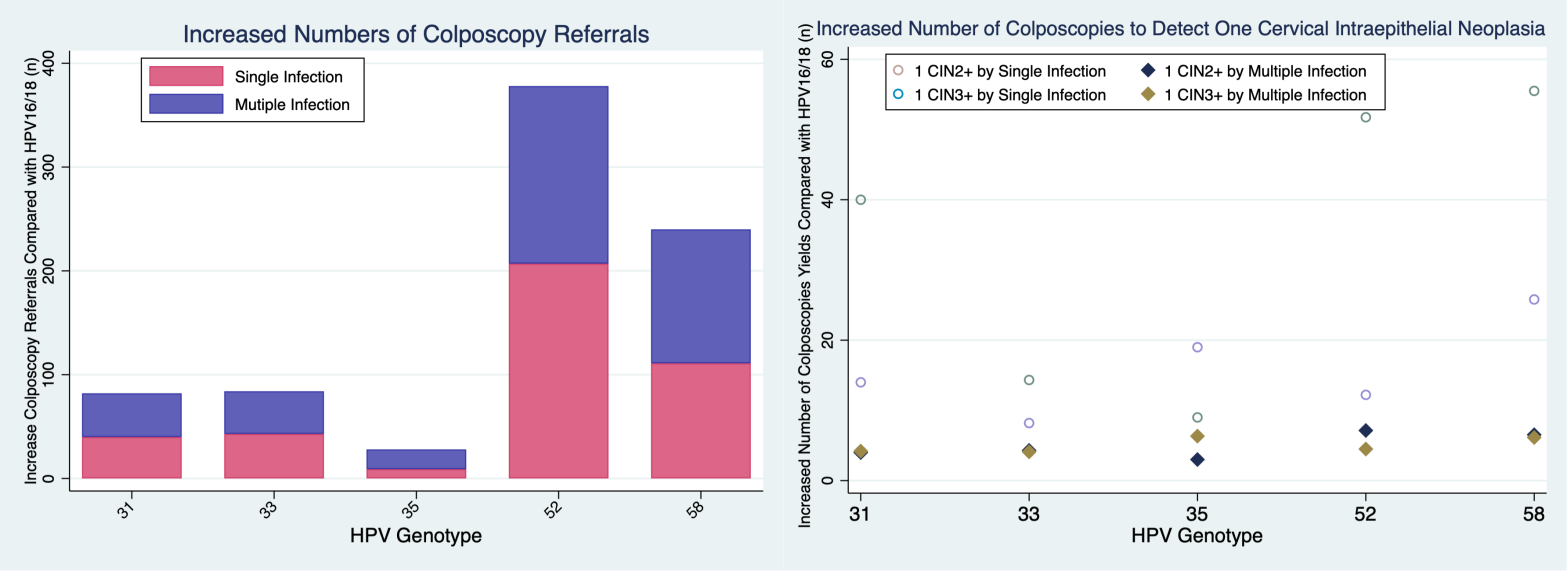
Diagnostic efficiency of non‐16/18‐hrHPVs genotyping models. *Notes*: Colposcopies to detect 1 CIN2+/CIN3+ were calculated as additional number of the histologically‐confirmed CIN 2+/CIN3+ divided by the increased number of colposcopies performed.

The strategy of triaging HPV16/18 with multiple HPV52 genotyping yielded the highest rate of CIN2+/CIN3+, which was more than 3‐fold that of detection from multiple HPV 31/33/35 infection (Figure 3). Figure 4 shows the diagnostic efficiency of non‐16/18‐hrHPVs genotyping models in detecting underlying CIN2+/CIN3+. Of all triage alternatives among HPCN women, those infected with HPV16/18 and single HPV52 were associated with the highest proportion of colposcopy referrals (40.86%). The colposcopy referrals rate for multiple HPV31 infection was 35.52%, for multiple HPV 33 infection was 35.49%, similar to the guidelines of referring HPV16/18 (34.16%). Multiple HPV58 genotyping also yielded increased detection of CIN2+; however, the strategy of triaging HPV16/18 and multiple HPV58 required 26 colposcopies to detect 1 CIN3+, compared with 12 required by multiple HPV52. The strategy of triaging HPV16/18 and multiple HPV33 only required 8 colposcopies to detect 1 CIN3+ and 4 colposcopies to detect 1 CIN2+, being the most cost‐effective.

## 4 DISCUSSION

Our study provided sufficient data on managing HPCN women with non‐16/18 hrHPV genotypes, and functioned as a vital guidance for the prevention and control of cervical cancer particularly in resource‐deprived areas. We highlight the significance of HPV31/33/35/52/58 in CIN2+/CIN3+, indicating that current triage guidelines relying on cytology testing may miss high‐grade cervical lesions. To minimize unnecessary colposcopy referrals, we evaluated different strategies and demonstrated that incorporating multiple HPV 31/33/52 infections into the previously recommended HPV16/18 triaging strategy can improve the identification of CIN2+/CIN3+ risks.

The prevalence of certain HPV genotypes largely determine the pathogenicity, making it crucial for identifying populations at risk of higher levels of cervical lesions.^18^ Our results were similar to another two studies^19^, ^20^ in China, reporting that the four most prevalent HPV genotypes were HPV16/52/58/18. The top four genotypes besides HPV 16/18 were HPV52/58/51/39 among the HPCN population, inconsistent with the four most common HPV types among the normal population from 38 countries (HPV16/18/31/33).^21^ Our results further highlighted the prevalent HPV 52 (12.23%) /58 (9.51%) among cytology‐negative women in China. Although an HPV positive result alone does announce an increased risk of cervical precancerous lesions and cancer, not all HPV genotypes are equally likely to persist or develop into precancer or cancer.^22^, ^23^

To avoid overburdening healthcare systems and over‐referral to colposcopy, HPV genotyping could be used to stratify the risk of HPV‐positive women.^24^ Our study found that the rate of single and multiple HPV 52 infections was higher among CIN3+ patients, consistent with previous studies.^19^, ^25^ Our study further revealed that HPV 52/58 pose a high risk of high‐grade cervical lesions similar to HPV16/18. In particular, single HPV 58 infection showed 1.74 times higher odds of CIN 2+, and multiple HPV52 infection showed 1.79 times higher odds of CIN2+ and 1.99 times increased odds of CIN3+ among HPCN women. HPV 45 also poses a high risk for CIN2+ but did not significantly increase the odds of CIN3+. However, CIN3+ is generally preferable as many CIN2s regress spontaneously.^26^ Notably, the risk of CIN2+/CIN3+ was not observed among women infected with HPV18, possibly due to its low prevalence and the short follow‐up period in this study. The prevalence of HPV18 was 3.31% in single cases and 4.19% in multiple cases among HPCN women in our study, slightly lower than the prevalence range (4.32%–5.24%) reported in a meta‐analysis of 8343 patients with CIN in China.^27^ A recent meta‐analysis showed that HPCN women had on average a 6.4% risk of CIN3+ within 5 years, but the 1‐year risk of CIN3+ was less than 4%.^28^, ^29^ Furthermore, previous studies indicate that HPV 18 carries the highest relative risk only for adenocarcinoma.^30^, ^31^ These findings provide a basis for adding HPV genotyping into primary screening using real‐world data.

HPV 31/33/35 and HPV 52/58 are consistently associated with higher risk for high‐grade cervical lesions than other hrHPV types in HPCN women. Among these, HPV 35 is related to 4.80 times higher odds of CIN3+, exceeding the immediate referral threshold in the latest ASCCP guidelines.^29^, ^32^ Our findings align with previous studies, such as Kjær et al.^33^'s identification of HPV18/31/33/58 as high‐risk types in HPCN women and Zhang et al.^34^'s report on the association between HPV 33 infection and histological HSIL+. Tjalma et al.^35^, ^36^ found that HPV 16/33/31 were most common among women with high‐grade CIN, and Cuzick et al.^30^, ^36^‘s research supported that HPV33 and 31 carried higher risks than HPV 18 for CIN3+. However, results on the clinical performance of non‐16/18 hrHPV infection should be interpreted with caution due to the limited number of cases. Although HPV 31/33/52/58 were considered to have higher risks, they are not sensitive enough to be the sole screening test. Nonetheless, their PPVs were higher than remaining high‐risk types, which makes them useful in determining the need for immediate colposcopy or repeat testing at a 6‐ or 12‐month interval.^37^

Efforts to determine the diagnostic values of different HPV genotypes for triage purposes have been made, but detecting a specific genotype cannot distinguish between a transient infection and a prevalent precancer.^38^ Our study found that the specific genotypes had AUCs of only around 0.5. Nevertheless, the costs of follow‐up colposcopy and false‐positive treatment may be offset by preventing certain cancers.^14^, ^39^ According to the 2019 ASCCP guidelines,^32^ immediate colposcopy referral is necessary for HPV‐positive women with LSIL cytology results and a CIN3+ risk of over 4.0%. Immediate colposcopy referral is recommended for HPV52/31/33/35 due to their high carcinogenicity and prevalence.^40^ Referring cytology‐negative women infected with single HPV 52 for colposcopy increased CIN2+ and CIN3+ detection compared to other genotypes. To reduce missed CIN3+ detection, colposcopy examination is recommended for patients infected with HPV 52/31/33. Immediate colposcopy is also recommended for women with HPV 35/58 infection if resources are sufficient. Our study found that only 12 colposcopies were needed to detect one CIN3+ case among women infected with multiple HPV 52 genotypes, compared to higher numbers required for other genotypes.^41^

Moreover, we observed that multiple non‐16/18‐hrHPV infections increased the odds of CIN2+ (OR = 2.95 95% CI = 2.40–3.62) and CIN3+ (OR = 4.39 95% CI = 3.11–6.20) than multiple HPV16 infections (OR = 2.29 for CIN 2+ and 3.95 for CIN3+) among HPCN women. The existing evidence on interactions between co‐infecting HPV genotypes is contradictory.^42^ Some studies show that multiple HPV infections act independently and have no additive effect on cervical disease compared to a single HPV infection,^27^, ^43^, ^44^, ^45^ while others suggest a cooperative interaction among multiple non‐16/18‐HPV genotypes in HPCN women.^46^, ^47^ Our findings could guide healthcare authorities in assessing vaccination programs and suggest the need for tailored HPV vaccines in China. The quadrivalent HPV (HPV16/18/6/11) vaccine has been shown to be highly effective in preventing CIN2+ and CIN3+ lesions,^48^ and the 9‐valent‐HPV vaccine can prevent high‐grade lesions and cervical procedures related to HPV 31, 33, 45, 52, and 58.^49^

Our study has several strengths. First, this study is the most geographically comprehensive study on the diagnostic value of non‐16/18 HPV genotypes, employing real‐world data from China, which allows for a better assessment of each type's relative importance. Second, we followed standardized clinical procedures, including immunohistochemistry for ambiguous diagnoses, blinded readings by pathologists, and consistent HPV typing assays across all samples. Nonetheless, some limitations need to be acknowledged. First, retrospective studies are constrained by available data. Future large‐scale studies with long‐term follow‐up could provide a more detailed and dynamic assessment of cervical cancer risk.^24^ Second, our study population comprised women with abnormal cervical screening results from different hospitals, not randomly selected, which might overestimate the diagnostic value of non‐16/18 HPV genotypes in cervical cancer. Moreover, our HPV results would be influenced by DNA source and detection techniques,^5^ which limits generalization to other countries. Lastly, additional studies are needed to elucidate the molecular mechanisms of HPV infection and carcinogenesis.

## 5 CONCLUSION

In conclusion, our study revealed that the most common HPV genotypes in China were HPV 16/52/58/18, and recommended women who test positive for HPV 16/18/31/33/35/52/58 directly to colposcopy, regardless of their cytology status. These findings have implications for countries with comparable settings to China and offer valuable insights into vaccine strategies. However, it is worth noting that further large‐scale population trials across diverse regions and settings are needed to comprehensively assess HPV genotyping's clinical performance before its global adoption into clinical practice.

## AUTHOR CONTRIBUTIONS

**Anying Bai:** Formal analysis (equal); methodology (lead); software (equal); visualization (lead); writing – original draft (lead); writing – review and editing (lead). **Peng Xue:** Conceptualization (lead); data curation (supporting); formal analysis (supporting); project administration (lead); supervision (equal); writing – review and editing (equal). **Qing Li:** Data curation (equal); investigation (equal); project administration (equal); resources (equal); supervision (equal); writing – review and editing (equal). **Yu Jiang:** Conceptualization (equal); funding acquisition (lead); project administration (supporting); resources (equal); supervision (lead); writing – review and editing (equal). **Youlin Qiao:** Conceptualization (equal); funding acquisition (equal); project administration (equal); resources (lead); supervision (equal); writing – review and editing (equal).

## FUNDING INFORMATION

This work was supported by Discipline Construction Project of School of Population Medicine and Public Health, Chinese Academy of Medical Sciences and Peking Union Medical College, and supported by CAMS Innovation Fund for Medical Sciences (CAMS 2021‐I2M‐1‐004).

## CONFLICT OF INTEREST STATEMENT

The authors have no conflict of interest to declare.

## ETHICS APPROVAL AND CONSENT TO PARTICIPATE

This study was conducted in accordance with the Declaration of Helsinki and received ethical approval from the Institutional Review Board (IRB) at the Chinese Academy of Medical Sciences and Peking Union Medical College (CAMS/PUMC).

## Supporting information


Table S1.

## Data Availability

The data generated in the present study may be requested from the corresponding author.
