# Supplementary material for: Diagnostic value of high‐risk HPV other than type 16/18 in high‐grade cervical neoplasia among cytology‐negative women: A multicenter retrospective study
Source: Cancer Med. 2023 May 18;12(13):14794–805. doi: 10.1002/cam4.6109 (PMC10358197; doi:10.1002/cam4.6109)
Supplement: Supplementary file 1 — Table S1. [file CAM4-12-14794-s001.docx]

**Table S1 Baseline characteristics**

|  | NILM(n=3,091) | ASC-H(n=286) | ASC-US(n=1,981) | HSIL(n=591) | LSIL(n=1,094) |
| --- | --- | --- | --- | --- | --- |
| **Age** |  |  |  |  |  |
| Mean (SD) | 38.7(10.3) | 42.8(9.7) | 39.0(10.4) | 42.3(10.4) | 38.8(9.8) |
| **Region (Centers)** |  |  |  |  |  |
| Shanxi | 725(23.46%) | 40(23.46%) | 368(18.58%) | 175(29.61%) | 282(25.78%) |
| Sichuan | 273(8.83%) | 273(8.83%) | 89(4.49%) | 12(2.03%) | 28(2.56%) |
| Gansu | 213(6.89%) | 213(6.89%) | 679(34.28%) | 89(15.06%) | 329(30.07%) |
| Jiangxi | 279(9.03%) | 279(9.03%) | 115(5.81%) | 52(8.80%) | 69(6.31%) |
| Shandong | 866(28.02%) | 866(28.02%) | 326(16.46%) | 192(32.49%) | 198(18.10%) |
| Guangdong | 735(23.78%) | 735(23.78%) | 404(20.39%) | 71(12.01%) | 188(17.18%) |
| **Gravidity** |  |  |  |  |  |
| Mean (SD) | 2.2(1.6) | 2.7(1.4) | 2.3(1.5) | 2.6(1.5) | 2.5(1.6) |
| **Parity** |  |  |  |  |  |
| Mean (SD) | 1.3(0.9) | 1.5(0.8) | 1.3(1.0) | 1.6(1.0) | 1.4(1.0) |
| **Menopause** |  |  |  |  |  |
| No | 2660(86.06%) | 224(78.32%) | 1703(85.79%) | 481(81.39%) | 957(87.48%) |
| Yes | 431(13.94%) | 62(21.68%) | 278(14.03%) | 110(18.61%) | 137(12.52%) |
| **Histopathology** |  |  |  |  |  |
| < CIN 2 | 2672(86.44%) | 108(37.76%) | 1607(81.12%) | 164(27.75%) | 792(72.39%) |
| CIN2+ | 298(9.64%) | 99(34.62%) | 258(13.02%) | 173(29.27%) | 213(19.47%) |
| CIN3+ | 121(3.91%) | 79(27.62%) | 116(5.86%) | 254(42.98%) | 89(8.14%) |
